# DNA methylation temporal profiling following peripheral versus central nervous system axotomy

**DOI:** 10.1038/sdata.2014.38

**Published:** 2014-11-11

**Authors:** Ricco Lindner, Radhika Puttagunta, Tuan Nguyen, Simone Di Giovanni

**Affiliations:** 1 Laboratory for NeuroRegeneration and Repair, Center for Neurology, Hertie Institute for Clinical Brain Research, University of Tuebingen, 72076 Tuebingen, Germany; 2 Center for Biotechnology and Biomedicine (BBZ), Division of Molecular Biological-Biochemical Processing Technology, University of Leipzig, 04103 Germany; 3 Molecular Neuroregeneration, Division of Brain Sciences, Imperial College London, Hammersmith Campus, W12 ONN, London

## Abstract

The regulatory mechanisms responsible for the gene expression pattern associated with axotomy-dependent signaling affecting the neuronal phenotype, including the axonal regenerative program, remain unclear. To further this understanding, we recently performed DNA methylation temporal profiling in lumbar dorsal root ganglia (DRG) after axotomy of the central spinal (non-regenerating) and of the peripheral sciatic nerve (regenerating) axonal branches. DNA methylation microarrays for mouse gene promoters and CpG islands (Roche/NimbleGen) were employed after immunoprecipitation of 5-methylcytosine-DNA. Here we provide a detailed data descriptor of this DNA methylation dataset, which allows in depth evaluation of the experimental design, assessment of data reproducibility and a full interactive operator-based systematic data analysis. In fact, we offer a methylation ‘hit’ scoring map of the whole microarray data in a workable spreadsheet that allows data sorting by genes, conditions or hits of interests that is ready for functional gene annotation and classification. This dataset allows investigators bioinformatic comparison to other epigenetic and gene expression datasets and further experimental characterization of the role of DNA methylation in axotomy-dependent pathways.

## Background & Summary

The purpose of this study was to elucidate the role of DNA methylation in the axotomy-dependent gene expression programme, including in the regulation of genes associated with cell metabolism, atrophy, survival and axonal regeneration in the pseudounipolar dorsal root ganglia (DRG) following peripheral sciatic nerve axotomy (SNA) compared to central dorsal column axotomy (DCA). Following nerve lesion, the DRG exhibits various degrees of cellular atrophy and cell death as well as an intrinsic regenerative response of the peripheral axonal branch. In stark contrast, the central branch within the spinal cord does not spontaneously regenerate when the lesioned axon is exposed to the local inhibitory environment^1^. Neuronal cell body phenotypic changes in response to axotomy are associated with specific patterns of gene expression, including the presence or absence of a regenerative gene expression pattern. Since DNA methylation is a key epigenetic mechanism responsible for the control of gene expression^2^, we systematically investigated the temporal regulation of DNA methylation on gene promoters in DRG after equidistant peripheral or central spinal axotomy.

According to our hypothesis, injury dependent differential changes in gene expression patterns were expected to be associated with corresponding changes in DNA methylation -DNA HYPOmethylation is associated with gene activation, and HYPERmethylation with gene repression-, including genes involved in cell metabolism, atrophy, survival, axonal transport and differential regenerative response.

This dataset (Data Citation 1), which we recently reported supplementarily in Puttagunta *et al.*^3^ included a genome-wide microarray analysis of DNA methylation patterns at gene promoters and CpG islands, following a methylated DNA immunoprecipitation approach (MeDIP-chip). Genomic DNA of dissected DRG was analyzed 1, 3 or 7 days following either sciatic nerve axotomy (SNA), a peripheral injury, or dorsal column axotomy (DCA), a central injury of the spinal cord. Mouse DRG were used as a suitable *in vivo* model for axon regeneration that allows the investigation of differential responses from either type of nerve lesion within the same neuron. A CpG island analysis of genes and their promoters allowed correlations between the normalized CpG dinucleotide distribution and injury-induced changes of promoter methylation or gene expression, measured by real time PCR in a subset of genes^4^.

Altogether, the most stringent data analysis identified 179 hyper- or hypomethylated genes for both injury conditions in all individual biological replicates. A subset of these genes (46) was differentially methylated (DM) exhibiting injury-induced changes of methylation levels only upon SNA or DCA. Additionally, we reported that many of these genes were associated with functions in chromatin remodelling, transcriptional regulation, axonal transport or neural development and differentiation. For a subset of the DM genes, the promoter methylation status correlated with gene expression changes upon injury in accordance with our hypothesis^3^ (Supplementary Figs 1 and 2 of Puttagunta *et al.*^3^). Gene expression of known major regeneration-associated genes (RAGs) such as *Gap-43*, *Galanin*, *Sprr1a* and *Bdnf* was verified to be upregulated solely upon SNA (of Puttagunta *et al.*^3^), however, none of these genes were significantly methylated (Supplementary Fig. 2 of Puttagunta *et al.*^3^) DM genes exhibited a higher normalized CpG density around the transcription start site (TSS) than RAGs, and differentially hypermethylated genes had higher normalized CpG values than hypomethylated genes as well as moderately induced RAGs had higher values compared to highly induced RAGs. It must be noted that this DNA methylation dataset does not cover the whole genome, but rather the proximal promoter regions of more than 18.000 genes, with a bias towards CpG islands (CGIs), mostly associated with gene promoters.

Here, the methods description is expanded from the description in our previous work (Supplementary Fig. 1, Puttagunta *et al.*^3^) NCBI's Gene Expression Omnibus GSE55514 (2014)) to include the entire dataset, the details of the design employed and the data analysis that has not been presented previously, which strongly limited the utility of the DNA methylation data as a whole.

Therefore, we aim here to provide the scientific community with a detailed methodological description of DNA methylation data as well as a user friendly access to the large temporal dataset mapping DNA methylation after regenerative versus non-regenerative axotomy. This is of importance as *(a)* this fully accessible DNA methylation dataset may contribute to the molecular understanding of gene regulation after axotomy as a whole, including the role of DNA methylation in neuronal metabolism, survival and axonal transport. Moreover, *(b)* it represents a useful as well as novel platform for the comparison of these data with other gene expression and epigenetic-based datasets, including the possibility to use these data to develop independent experiments aimed at testing axonal injury-related pathways.

In summary, we believe the present data descriptor will allow a direct and ‘hands on’ assessment of epigenetic regulation of gene expression post-axotomy, and will permit novel insight into the role of selected differentially methylated genes in gene regulatory mechanisms responsible for axotomy-dependent changes affecting the neuronal phenotype.

## Methods

### Animal model and surgery

All mice used for this work were treated according to Animal Welfare Act and to the ethics committee guidelines of the University of Tuebingen. C57BL/6 wild type mice (Charles River Laboratories International) were used for all experiments presented here, aged from 6 to 12 weeks. Any treatment or surgery was performed in a way to avoid stress as much as possible. All surgical procedures were performed under aseptic technique and general anesthesia. For surgeries, mice were anesthetized with ketamine (100 mg/kg body weight) or with xylazine (10 mg/kg body weight), and with Isofluran/O_2_ (Isoba®; initially 5 percent, maintained at 2 percent). Either sciatic nerve axotomy (SNA), or dorsal column axotomy (DCA), or the corresponding sham injury was performed on different sets of mice. Another set of naive mice received no surgery.

### Sciatic nerve axotomy

Mice were anesthetized, and bilateral surgery was successively performed. At a distance of approximately 20 mm from L4-L6 DRG, a 10 mm skin incision was performed on the gluteal region at mid-thigh level. Muscles were displaced to expose the sciatic nerve for a complete transection with spring micro-scissors. Finally, skin was closed with two suture clips. The nerve fiber was left intact for sham surgeries; otherwise the same procedure was applied for all samples.

### Dorsal column axotomy

Mice were anesthetized. Surgeries were performed as previously reported^5^. A T10 laminectomy was performed, approximately 20 mm from L4-L6 DRG. An incision from T7 to T10 was made and superficial tissue displaced or carefully removed. Holding the spinous process, the side connecting bone was cut and the top half of the vertebrae was lifted away. A few drops of Xylocain were applied to anesthetize the spinal cord. Then, the dura mater was removed taking care of not damaging the spinal cord. A bilateral dorsal hemisection until the central canal (0.3 to 0.4 mm depth) was performed with a micro-knife. For the control laminectomy surgery, the dura mater was removed but the dorsal hemisection was not performed. Finally, skin and tissue were closed with two suture clips.

### DRG dissection and sample preparation for MeDIP

For each of the 3 time points and injury conditions (1, 3, 7 days after SNA or DCA, injury or sham, and naive), L4-L6 DRG were collected from 2 mice and pooled for each individual sample (in triplicate for injury and naive, and in duplicate for sham). Animals were deeply anesthetized and killed by cervical dislocation. The spinal cord and DRG were exposed from the ventral side, DRG were quickly dissected, kept in HBSS buffer on ice, cleaned and frozen in liquid nitrogen. Frozen tissue was ground, lysed in 200 μl MeDIP lysis buffer and digested with 0.2 mg/ml Proteinase K overnight at 50 °C and shaking at 400 rpm. The lysate was then sonicated on ice to a chromatin size range of 100 to 2,000 bp, with 700 bp average (Bandelin Sonopuls GM70, type UW 70 sonication device with micropestle; 6 times, 10 s, 50% pulse, 30% power). Genomic DNA was extracted from the cleared lysate by standard phenol-chloroform extraction and ethanol precipitation procedures. Sonication efficiency was optimized and tested on agarose gel before performing Methylated DNA immunoprecipitation (MeDIP). DNA concentration and quality was assessed with a peqlab NanoDrop ND-1000 as well as on an agarose gel.

### Methylated DNA immunoprecipitation

**Figure 1:**
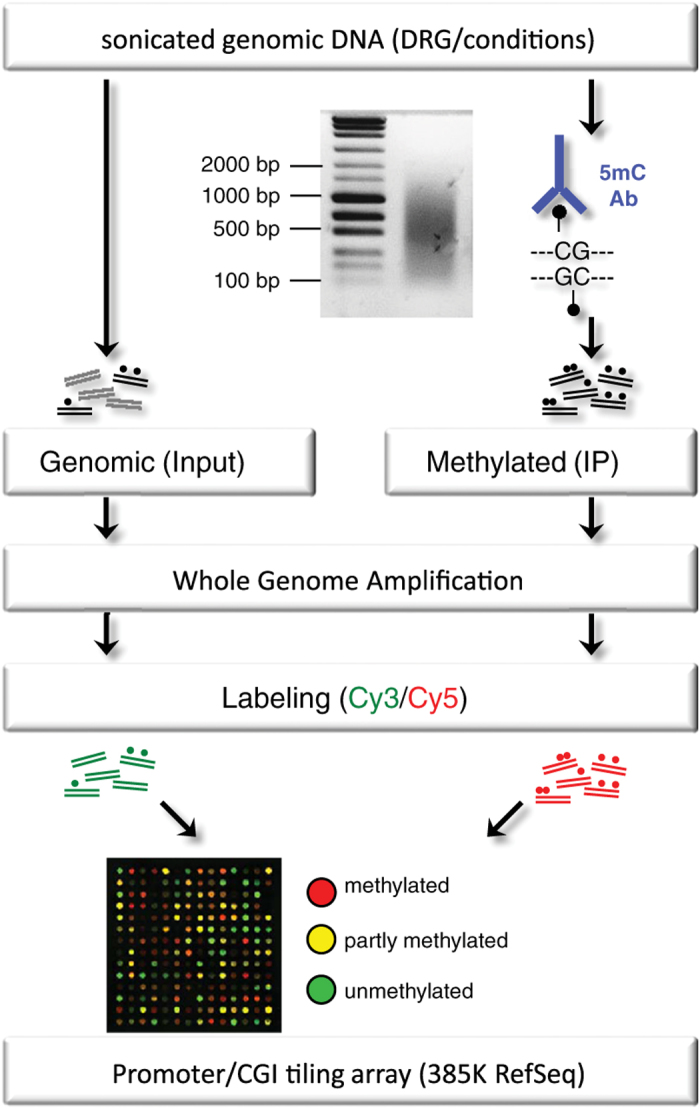
Schematic of MeDIP-chip procedure. Sonicated genomic DNA was immunoprecipitated (IP) with a 5-methylcytosine antibody. IP samples (methylated fragments) and genomic DNA (Input control) samples were amplified supposedly up to 1,000-fold with a Whole Genome Amplification (WGA) Kit (Sigma-Aldrich) and purified. Each corresponding set of IP and Input sample libraries were conjugated with either Cy5 or Cy3, respectively, and co-hybridized on a DNA methylation sensitive tiling microarray (Roche/NimbleGen). Relative IP-to-Input enrichment of methylated DNA fragments was verified by PCR.

MeDIP procedure, subsequent Whole Genome Amplification (WGA), and sample preparation for Roche/NimbleGen DNA methylation microarrays (see Fig. 1) were performed according to a modified protocol adapted from Komashko *et al.* and, in part, from Weber *et al.*^6,7^ Specifically, MeDIP was performed according to the protocol of the Chromatin Immunoprecipitation (ChIP) Kit from Upstate/Millipore. 10 μg of sonicated purified genomic DNA were added to a total volume of 150 μl of ChIP SDS lysis buffer, denatured for 10 min at 95 °C, and quickly chilled on ice. Samples were then diluted 10-fold in ChIP Dilution Buffer. To reduce nonspecific background, diluted samples were pre-incubated with 75 μl of Protein A/G agarose beads (50 percent slurry with salmon sperm DNA) and incubated for 30 min at 4 °C with agitation. The supernatant was incubated with 5 μg of a 5-methylcytosine antibody (Eurogentec, BI-MECY-0100) to immunoprecipitate methylated DNA fragments overnight, with agitation at 4 °C. A no-antibody negative control was used in parallel.

Antibody-DNA complexes were incubated with 60 μl of Protein A/G agarose beads for 1 hour at 4 °C with agitation. Beads were briefly pelleted and the supernatant kept at −20 °C. Immune complexes were successively washed with Low Salt, High Salt, LiCl, and twice with TE Wash Buffer followed by elution of the DNA/antibody/bead complexes by adding twice 250 μl of freshly prepared Elution Buffer and incubating for 15 min at RT under agitation. The eluted complexes were digested with 0.1 mg/ml Proteinase K for 1 h at 45 °C with agitation after adding 10 μl of 0.5 M EDTA and 20 μl of 1 M Tris/HCl (pH 6.5) to 500 μl elute. DNA was then recovered from the IP or no-antibody control samples by standard phenol/chloroform extraction and ethanol precipitation (EtOH/NaOAc/glycogen). Finally, samples were resuspended in 40 μl TE buffer. Regular PCR was employed to test MeDIP efficiency prior to performing the real MeDIP-chip experiments upon nerve injury. IP and Input samples from naive mice were tested for 4 primer sets targeting the methylated *H19* imprinting control region (ICR)^8^. Primer sequences for *H19* ICR and *Actin* as negative control were obtained from Komashko *et al.*^7^

### Whole genome amplification

Due to the low IP sample yield in the range of a few hundred nanograms, the GenomePlex Complete Whole Genome Amplification (WGA) Kit (#WGA2, Sigma-Aldrich) was performed to amplify 20 ng of IP and Input samples to a maximum yield of 3 to 7 μg. Representation analysis has been verified before in order to prove representative amplification with minimal sequence bias^9,10^. Since in this study genomic DNA was already fragmented due to sonication, the first step of the WGA kit was modified to eliminate the fragmentation step. IP or Input samples were amplified, together with a positive Human Control DNA sample to verify amplification success. According to the manufacturer’s protocol, Fragmentation Buffer, Library Preparation Buffer, and Library Stabilization Solution were added to the sample. The reaction mix was incubated for 2 min at 95 °C, cooled on ice, and Library Preparation enzyme was added. Library formation was achieved with the recommended thermocycler program: 1. 20 min at 16 °C, 2. 20 min at 24 °C, 20 min at 37 °C, 5 min at 72 °C, and holding 4 °C. Further, Amplification Master Mix, nuclease-free water, and WGA DNA Polymerase were added. The complete reaction mix was amplified according to the following thermocycler program: 1. 3 min at 95 °C, 20 cycles of: 2. 15 sec at 94 °C, 3. 5 min at 65 °C, and holding 4 °C. Samples were subsequently column-purified, as recommended, using the GenElute PCR Clean-Up Kit (Sigma) to remove residual nucleotides and fragments smaller than 100 to 200 bp, according to the manufacturer’s protocol. DNA concentration and quality were assessed with a peqlab NanoDrop ND-1000. Samples were adjusted to a concentration of 250 ng/μl in order to meet Roche/NimbleGen’s instructions for DNA sample quality (see ‘NimbleChip Arrays User’s Guide for DNA Methylation Analysis’). For all samples, optimal ratios were obtained for DNA purity (A_260_/A_280_=1.92±0.04 and of A_260_/A_230_=2.30±0.05).

### DNA methylation microarray

Preparation of MeDIP-WGA samples for the DNA methylation microarray analysis was described before^7^. 5 μg of each WGA sample (triplicate or duplicate sample sets of IP and Input for each condition) were sent to the Roche/NimbleGen facility for DNA methylation sensitive tiling microarrays. Briefly, for each microarray a corresponding pair of IP and Input samples (1 μg) were labeled with Cy5 and Cy3, respectively, and co-hybridized on a ‘2007-02-27 MM8 CpG Island Promoter (385 K RefSeq)’ tiling microarray. This array type covered the proximal promoter regions of more than 18.000 genes, and CpG islands (CGIs) mostly associated with gene promoters. Genes and promoters were thus represented by regions of approximately 1,500 bp upstream and 800 bp downstream of a transcription start site (TSS) covered by several close-set oligonucleotide probes. Additionally, promoter and non-promoter CGIs were represented on the microarray. Fluorescence intensity raw data was obtained from scanned images of the tiling arrays using NimbleScan extraction software. For each spot on the array, Cy5/Cy3 ratios were calculated and normalized to obtain log2 values for enrichment. Then, the bi-weight mean of log2 values for each region was subtracted from each data point to center the ratio data to zero, similar to a mean-normalization. Significant enrichment in a walking 200-750 bp window was assessed using a Kolmogorov-Smirnov test yielding a series of log10 *P*-values. In the case of an average log10 value higher than 2 within the window, this peak value marked the significant methylation event at a promoter/CpG island. For subsequent global methylation analysis and for the comparison of methylated genes between conditions, an unpaired two-tailed *t*-test was applied. Significance is given as *P≤0.05, **P≤0.01, ***P≤0.001.

### Quantitative real-time RT-PCR-RNA extraction and cDNA synthesis

Total RNA was extracted and pooled from dissected L4-L6 DRG of 3 mice for each experimental condition applying the High Pure RNA Parafin Kit (Roche) following the manufacturer’s protocol. RNA concentration was determined with a peqlab NanoDrop ND-1000. cDNA was synthesized from 1 μg of total RNA using the SuperScript II Reverse Transcriptase kit (Invitrogen) with oligo(dT) primers, according to the manufacturer’s protocol. 5 μl of 1:25 diluted cDNA was used in qRT-PCR experiments using ABsolute QPCR SYBR^®^ Green ROX Mix (Thermo Scientific) on an ABI 7000 Real Time PCR System (ABS/Life Technologies).

### Custom TaqMan gene expression array

A qRT-PCR-based Custom TaqMan^®^ Gene Expression Array (ABS/Life Technologies) was applied to test injury-induced gene expression of differentially methylated (DM) genes from the microarray study. TaqMan primer sets (sense, antisense, and FAM-probe) for 38 DM genes were provided by the manufacturer, together with controls (18S RNA, *Actb*, *Gapdh*). For each gene, corresponding samples (SNA/DCA, injury/sham) were assayed for the specific time point of differential methylation using TaqMan^®^ GEx Master Mix on an ABI 7000 Real Time PCR System (ABS/Life Technologies). TaqMan primer sets (assays) were delivered in a lyophilized form in suitable 96-well plates arranged in a customized array pattern. Standard cycling parameters were applied for qRT-PCR: 1. 94 °C for 10 min, 2. 95 °C for 15 s, 3. 60 °C for 60 s (40 cycles for steps 2. to 4.), 5. holding 4 °C. Relative quantification of fold change expression ratios were calculated according to standard delta-ct method normalized to the *Actb* reference gene.

### Promoter CGI analysis

Several major RAGs and DM genes (from DNA methylation microarray analysis) were analyzed for CGI and CpG dinucleotide distribution. The complete genomic plus promoter region (5,000 bp upstream of the TSS) was obtained from the Ensembl genome browser database (www.ensembl.org) updated for 2011 entries. The identity of the major transcript was confirmed by the UniProtKB database (www.uniprot.org/uniprot/). Genomic sequences were analyzed with the EMBOSS CPGPlot online tool from EMBL-EBI, applying standard parameters: observed-to-expected (obs/exp) ratio>0.6, and (C+G) content>50 percent for at least 200 bp. In several cases, a CGI size of 100 bp was allowed. Multiple CGIs in very close proximity were combined to one large CGI. Additionally, the normalized CpG values were calculated as the obs/exp CpG ratio (Formula 1) for the symmetrical 3 kb region around the TSS of the selected RAGs and DM genes, according to Saxonov *et al.*^4^

(1)obs/expCpGratio=(Number of CpG⋅3kb)/(Number of C⋅Number of G)

### DNA methylation microarray analysis

For the 12 experimental conditions plus naive, altogether 33 data points/microarrays were analyzed for each gene. Sham surgeries, at least in the case of laminectomy, might cause an effect on gene expression similar to nerve injury^11^. A color code was defined to visualize the comparative MeDIP-chip analysis of SNA (green), DCA (red), or naive (blue).

**Figure 2:**
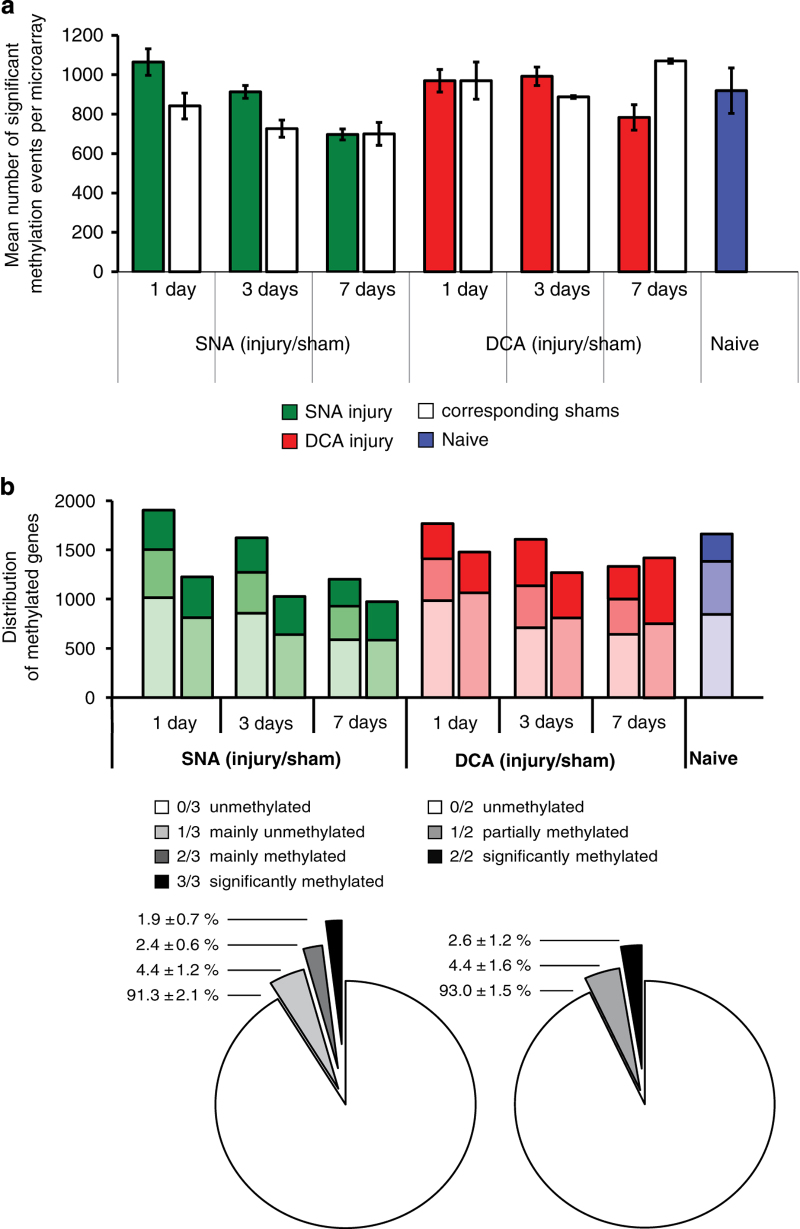
Global DNA methylation microarray analysis. (**a**) The total hit number of significant methylation events (mean) for all 18,180 annotated genes (by NCBI GeneID) was regarded as a measure for global methylation. The average total hit number for each multiple microarray set is graphed comparing SNA injury (green) or DCA injury (red) to corresponding shams (white), or naive (blue). Triplicate arrays were applied for injury conditions and naive, duplicates for sham. (**b**) The distribution of gene promoter methylation levels was compared between conditions (bar graph). Most genes were not significantly methylated for each condition, as shown as percentage range in pie charts. The numbers of thus methylated and partially methylated genes were compared (hitting in at least 1/3 arrays for injury or naive, or in at least 1/2 arrays for shams). The average percentage of methylated, partly methylated, or unmethylated genes was similar across all microarrays. The legend for grades of DNA methylation applies for both graphs. Error bars: s.e.; statistics: unpaired two-tailed *t*-test *P≤0.05, **P≤0.01, ***P≤0.001.

First, a global analysis approach was chosen to analyze changes of global DNA methylation between different experimental conditions. The total number of 18,180 genes annotated by their NCBI gene ID was the reference for the maximum hit number on one array^7^. The average number of significant methylation events (hits) of an array set for each condition represented the number of methylated genes (gene promoters), which was then interpreted as a semi-quantitative measure for global DNA methylation, without unit. Average global methylation levels were compared between conditions. These total numbers resulted either, from triplicate injury or naive array sets, or from duplicate sham sets, and are summarized in Fig. 2a. Second, the numbers of genes with specific ‘methylation levels’ were compared between experimental conditions and across the time course (Fig. 2b). Genes were regarded as ‘unmethylated’ if they were not significantly methylated in any of the arrays of a set (0/3 or 0/2), or as significantly ‘methylated’ if hitting in all arrays (3/3 or 2/2). The remaining genes were regarded as ‘partly methylated’ (1/3, 2/3 or 1/2). The qualitative nature of the pre-analyzed *P*-value dataset obtained from the Roche/NimbleGen microarrays does not allow for direct quantitative calculation of methylation levels. Comparison of changes upon SNA and DCA across the time course, as well as the combined numbers of methylated (3/3) and partially methylated genes (1/3 or 2/3) upon injury are reported in Fig. 2b. On average in all time points, similar numbers of methylated and partially methylated genes were identified for both injury types. However, it is of interest to note that following the time course, a clear downward trend for the numbers of methylated and partially methylated genes was observed only upon SNA injury, compared to corresponding shams. In contrast, the gene numbers were usually similar between DCA injury, DCA sham, and naïve. Regarding injury, sham, and naïve conditions separately, most gene promoters were not significantly methylated, which is consistent with previous findings for CpG island DNA methylation^6,7,12^. However, 1.5 to 3.8 percent of all genes were significantly methylated for a single condition (in all arrays). Regarding all conditions together (33 microarrays), 78.8 percent (14,325) of all genes remained unmethylated for all conditions whereas 0.2 percent (36 genes) showed significant methylation in every microarray across all conditions (Fig. 2b).

**Figure 3:**
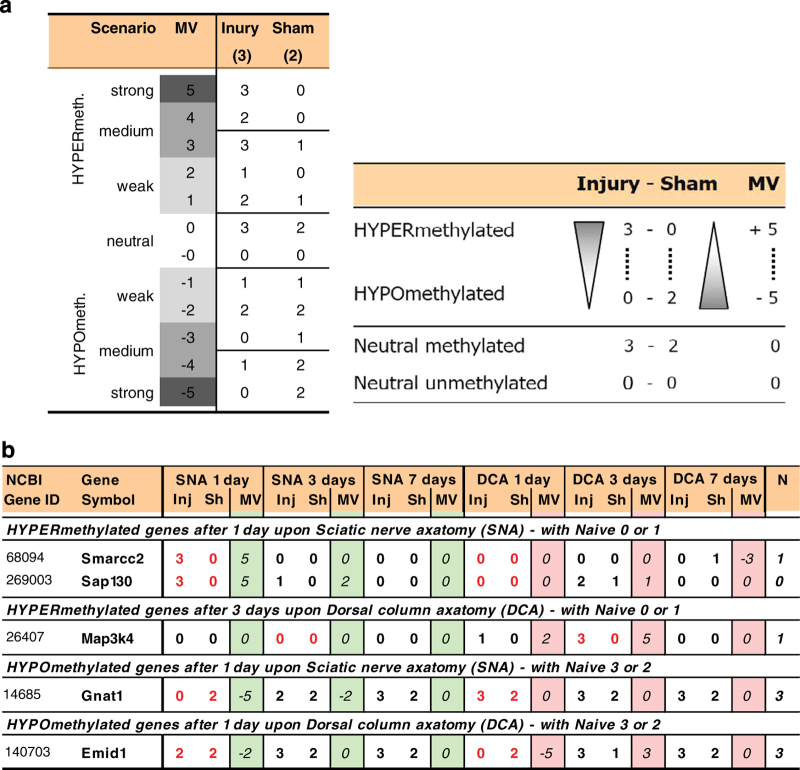
Demonstration of methylation hits compared to a new methylation value system. (**a**) Legend for defined Methylation Values (MV) and schematic for semi-quantitative analysis of the DNA methylation microarray dataset. Arbitrary MV were defined according to the ranking of calculated differences (D_MV_) between relative injury and sham hit numbers (see Text) in order to determine genes with strong, medium, or weak hyper- and hypomethylation for each condition (with respect to naive being similar to sham). (**b**) Selected differentially hyper- or hypomethylated genes are visualized as an example of filtering genes applying the supplemented interactive NimbleGen analysis table. These genes exhibit a different injury-induced promoter methylation state only upon one injury type while not changing upon the other, regarding combined injury (Inj) and sham (Sh) for each condition.

Specific scenarios and algorithms were also defined to filter potential genes of interest. First, hyper- or hypomethylated genes were identified for each condition. Hereby, ‘hypermethylated’ is deemed significantly methylated upon injury but not methylated in the corresponding sham. Consequently, ‘hypomethylated’ is defined as not being methylated upon injury but methylated in sham for a specific condition. It has to be noted that hyper- or hypomethylated genes were pre-filtered for appropriate methylation levels in naïve. This was done in order to eliminate uncertain candidates, thus respecting that sham values were supposed to be similar to naïve. The ‘Methylation Value’ (MV) was defined as a semi-quantitative measure to distinguish between strong, medium, or weak hyper- or hypomethylation or neutral cases (Fig. 3a). The arbitrary MVs were defined according to the ranking of calculated differences (D_MV_) between relative hit numbers for injury and sham array sets (Formula 2). These relative hit numbers describe the above mentioned levels full, partial or no methylation for a specific gene in a triplicate or duplicate array set. The D_MV_ values were then ranked from highest to lowest and assigned each to a number between +5 and −5 indicating strong hypermethylation down to strong hypomethylation, including neutral cases. For example, 2 out of 3 hits for injury and 0 out of 2 hits for sham (2-0) are calculated to an D_MV_ of 0.7 which was assigned to MV=+1 and medium hypermethylation (Fig. 2). Neutral cases for no change of the methylation status upon injury are assigned with an MV=‘M’ for genes that were methylated in shams and remain methylated upon injury. Vice versa, persistingly unmethylated genes were assigned with MV=‘U’. Both cases correspond to a calculated D_MV_=0 (MV=0).

(2)DMW=NumberofHits(injury)/3−NumberofHits(sham)/2

For a number of gene promoters, certain discrepancies for methylation levels were found across all shams and naive. That is why strict filters were applied for the main analysis in order to find genes that were clearest hyper- or hypomethylated at each single condition (Fig. 3a). Thus, the maximum positive MV=+5 stands for strong hypermethylation (injury: 3/3 hits; sham: 0/2 hits), and maximum negative MV=−5 for strong hypomethylation (injury: 0/3 hits; sham: 2/2 hits). Applying a strict analysis algorithm allowing only MV=+5/−5 and regarding naive values, required to be similar to shams, yielded sets of strong hypermethylated genes (with naive: 0/3 or 1/3) or strong hypomethylated genes (naive: 2/3 or 3/3) for each condition. These genes were then analyzed and classified for functional annotations as recently shown^4,13^.

Importantly, the formula above constituted the scientific basis for the generation of a user friendly workable excel spread sheet now made available here, where different criteria can be applied to DNA methylation data analysis (Supplementary File 1) based upon the user specific research question. In particular, users can select gene/s of interest and match it with specific methylation ‘hits’ (0 to 3) per each experimental condition (including, sham, SNA and DCA or Naive), and can select the time point post-axotomy of interest (1, 3 or 7 days). Additionally, we have created ‘interactive counters’ above each column that allows a quick quantification of genes with a specific methylation status (by MV, or by hit number for sham or injury) for each condition. Finally, the numbers above each column (in the green or red fields) are the total average hits of the microarray sets for each condition (representing global DNA methylation values). This analysis can be easily performed using basic Excel sorting functions and results can be pasted and employed for data sharing and/or for gene annotation and classification with online gene function algorithms such as DAVID (http://david.abcc.ncifcrf.gov/) or subscription based tools such as Genomatix or Ingenuity. The goal is to allow scientist novel discoveries by initiating projects based upon the identification of interesting genes and pathways differentially methylated after central versus peripheral axotomy.

## Data Records

The complete DNA methylation microarray data set, provided by Roche/NimbleGen, has been deposited in the NCBI Gene Expression Omnibus (GEO) database (Data Citation 1) in association with a Nature Communications publication^3^. The raw data set comprises of typical pair files (.txt), and the pre-processed data set consists of tab-delimited files (.xls), and NimbleGen SignalMap graphic files (.gff).

## Technical Validation

**Figure 4:**
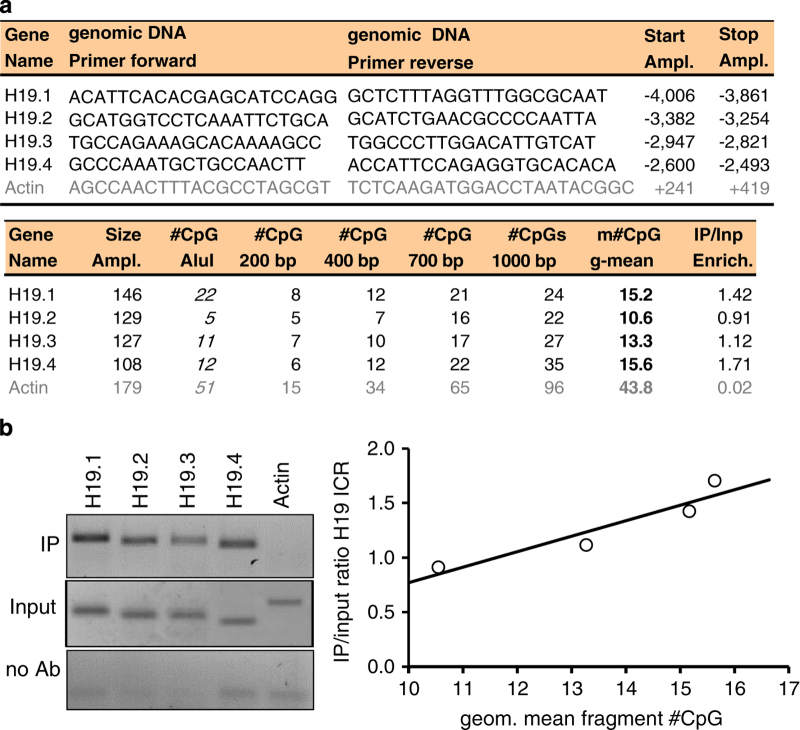
Verification of Methylated DNA Immunoprecipitation (MeDIP) on the *H19* Imprinted Control Region (ICR). To establish MeDIP on sonicated genomic DNA from naive mouse DRG, enrichment of 4 sequence fragments from the fully methylated *H19* ICR locus was measured. 10 μg of sonicated input genomic DNA (Input) and equal amounts of immunoprecipitated DNA (IP) or a no-antibody control (no Ab) were PCR amplified. (**a**) Primer sequences were selected from Weber *et al.* who used the AluI restriction enzyme to digest genomic DNA^6^. For all *H19* ICR sequences, the numbers of CpG dinucleotides are given for hypothetical sizes of sonication fragments around the center of each amplicon. (**b**) The graph displays the quantitative correlation between enrichment (IP/Input ratio), obtained by densitometry analysis of PCR bands in agarose gels, and the weighted geometric mean number of CpGs, which represents the population of fragments. An unmethylated *Actb* region as negative control was not enriched. No-antibody controls yielded no specific signals.

In preparation for MeDIP-chip experiments, the immunoprecipitation procedure from naïve DRG needed to be established. A modified MeDIP protocol was applied and adopted from Komashko *et al.*^7^ In pilot experiments, MeDIP was performed on 10 μg of genomic DNA from naive mouse DRG, sonicated to an average fragment size of 700 bp (100 to 2,000 bp) and incubated with a 5-methylcytosine antibody (IP) compared to a no-antibody control (no Ab). Additionally, genomic DNA (Input) was used as a reference sample. An initial verification of efficiency and specificity of the MeDIP protocol was performed with minor modifications according to the setup in Weber *et al.,* where the high sensitivity of the antibody was demonstrated allowing for a quantifiable analysis^6^. Therefore, four described PCR primer sets were used for the *H19* imprinting control region (ICR; amplicons 1 to 4) that is completely methylated^7,8^. This genomic region serves as quantifiable positive methylation control. In Weber *et al.*, genomic DNA was fragmented by the Alu1 restriction enzyme (cutting AG-CT) to yield defined fragments with distinct numbers of CpGs (21, 5, 11, or 12 CpGs for *H19* ICR 1 to 4). The more methylated CpGs are present in a sequence of interest, the more 5-methylcytosine antibody molecules should bind fragments representing this sequence, which will consequently be more enriched during immunoprecipitation. Unmethylated sequences should not be enriched. A primer set for the *Actb* promoter was used as negative control. The *Actb* housekeeping gene contains a large CpG island that is supposed to be largely unmethylated. Weber *et al.* used the AluI fragmentation only for the verification of the MeDIP. Otherwise, genomic DNA was sonicated for the following experiments following surgery, similar to this study. However, MeDIP verification in this work was performed on sonicated DNA as we planned to do for MeDIP-chip studies. Therefore, the alternative fragmentation method yielded different raw results compared to Weber *et al.* Quantification of enrichment for *H19* ICR sequences was adjusted to the weighted average of the sonicated fragment size range. Figure 4 displays the applied primer sequences and the positions of amplicons relative to the TSS of the *H19* locus. Also, the numbers of CpGs are given for either the AluI fragments (produced by Weber *et al.*), or for representative hypothetical fragments centered round the amplicons. The weighted geometric mean CpG number for each representative fragment was calculated from the numbers of CpGs for a given arbitrary fragment size (Formula 3) and correlated to the *H19* ICR enrichment ratios. The geometric mean was weighted because of the typical Gauss fragment size distribution of regular IP or whole genome amplified IP samples (200 to 1,000 bp) used afterwards for the microarrays analysis.

(3)Weighted#CpG=geometricmeanof(#CpG200bp⋅#CpG400bp2⋅#CpG700bp2⋅#CpG1,000bp)

**Figure 5:**
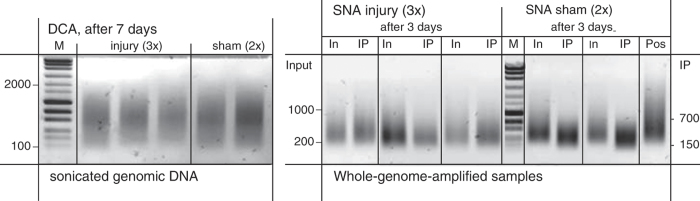
Quality control of whole-genome-amplified (WGA) samples after MeDIP for DNA methylation microarrays. Genomic DNA (Input for MeDIP) was extracted and pooled from each 2 adult mice for each sample of a condition, and sonicated to an average fragment size of approximately 700 bp (100 to 2,000 bp). For each injury condition (SNA/DCA, 3 time points) and naive, a triplicate of pooled samples was used, and duplicate sets for each sham condition. WGA samples displayed a smaller average fragment size of 400 bp (200 to 800 bp for Input, and 150 to 700 bp for IP). As positive control (Pos), non-fragmented commercial human genomic DNA, included in the kit, was whole-genome-amplified as well.

PCR was performed for IP, no-Ab, or Input samples and PCR amplicons were separated on agarose gel for quantitative densitometry analysis using the GelPro32 software. IP/Input enrichment ratios, deduced from PCR signal intensities, roughly correlated with the expected weighted average numbers of CpGs for the different *H19* fragments. The *Actb* negative control was not enriched, nor did the no-Ab controls yield any specific signals (Fig. 4). Also, following IP and WGA (see methods), fragment size distribution and sample quality was verified on agarose gel (Fig. 5).

In order to investigate the inter-array variability we have performed a Pearson’s correlation coefficient analysis. This analysis was generated by collecting all log2-values together and creating comparison diagrams of each array of a replicate set to obtain commonly used Pearson’s correlation coefficients (R), and their square values (R^2^), respectively. A good correlation between biological replicates was observed as documented in Supplementary File 2.

**Figure 6:**
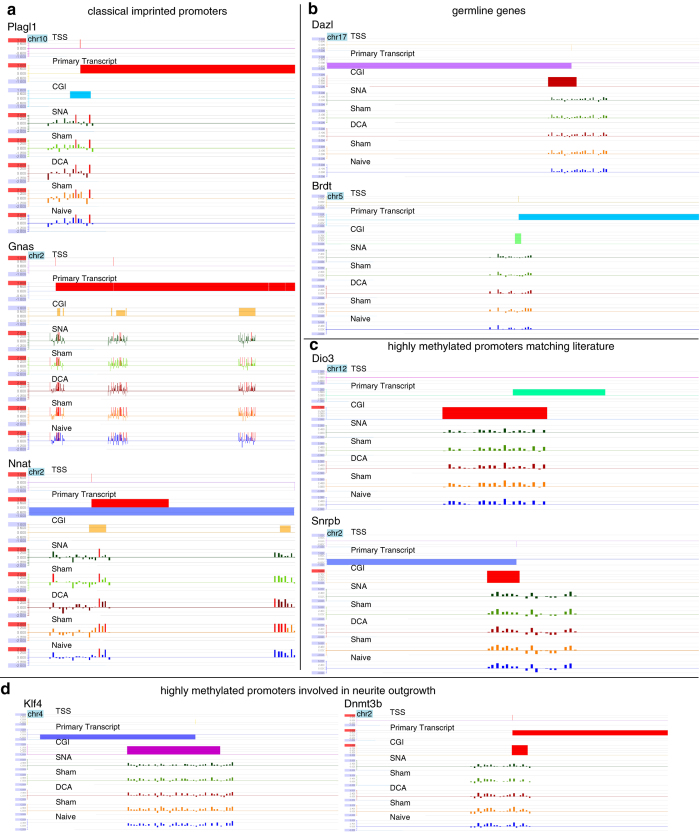
Signalmap snapshot views of methylated promoter regions. Snapshot views from Signalmap of methylated promoter regions show the transcription start site (TSS), primary transcript, CpG islands (CGI) and methylation data of these regions from the samples (SNA, Sham, DCA, Sham and Naïve) (Ensemble database 02–2007). The 5 tracks show the scaled and centered log2-values for each oligonucleotide probe (representing IP- to-Input enrichment ratios, normalized to the bi-weight mean methylation of the whole array), red bars in each track indicate *P*-values of greater + 2 or smaller − 2, which is supposed to be signiﬁcantly enriched for the speciﬁc probe (log2-values). (**a,b**) Classical imprinted promoters; Plag1 (12,777,600 to 12,784,100 bp), Gnas (173,923,000 to 173,986,000 bp) and Nnat (157,245,600 to 157,262,000 bp), as well as germline genes; Dazl (49,749,600 to 49,764,000 bp) and Brdt (107,564,400 to 107,577,900 bp) show validation that the array used here detects methylation in and surrounding CGI. Y-axis log2 scale from + 2 to − 2. (**c**) Dio3 (110,723,200 to 110,730,600 bp) and Snrpb (129,866,800 to 129,874,400 bp) are two representative methylated genes we observed in our array that are also found to be methylated in the literature in mice. Y-axis log2 scale from + 5 to − 3. (**d**) Klf4 (55,547,800 to 55,558,200 bp) and Dnmt3b (153,336,000 to 153,345,600 bp) are two examples of methylated CGIs that have been previously shown to be involved in neurite outgrowth. Y-axis log2 scale from + 5 to − 3.

Furthermore, in support of the quality of our methylation data, we found that several highly methylated genes in our arrays across conditions are validated or predicted-imprinted genes according to a current online repository (http://www.geneimprint.com/site/genes-by-species.Mus+musculus), while others match a recent high throughput analysis of the DNA methylation profile in neurons^14^ or show correlation to methylation in other conditions such as cancer^15^ (Fig. 6, Supplementary File 3). Interestingly, in line with our findings, Iwamoto and colleagues recently found that neuronal methylation events on CGI are limited with high inter-individual variation.

## Additional information

**How to cite this article:** Lindner, R. *et al.* DNA methylation temporal profiling following peripheral versus central nervous system axotomy. *Sci. Data* 1:140038 doi: 10.1038/sdata.2014.38 (2014).

## Supplementary Material

Supplementary File 1

Supplementary File 2

Supplementary File 3
